# Efficacy of lumbar epidural steroid injections for lumbosacral radiculopathy in individuals with obesity: A retrospective comparative study

**DOI:** 10.1016/j.inpm.2022.100109

**Published:** 2022-06-15

**Authors:** Ivan Chew, Rafael De Souza, Joan Reisch, Jonathan Le

**Affiliations:** University of Texas Southwestern Medical Center (Dallas, TX), 5323 Harry Hines Blvd, Dallas, TX, 75390, USA

**Keywords:** Lumbar radiculopathy, Lumbosacral radiculopathy, Lumbar epidural steroid injection, Transforaminal epidural steroid injection, Caudal epidural steroid injection, Interlaminar epidural steroid injection, Radiculitis, Obesity, Morbid obesity

## Abstract

**Purpose:**

Obesity, as defined by the CDC, is characterized by a body mass index exceeding 30. Over one third of the world's population is classified as overweight or obese. Furthermore, low back pain is currently the leading cause of disability worldwide. Patients who do not respond adequately to conservative therapy can consider more invasive procedures such as epidural steroid injections (ESI) as treatment for their low back pain. The purpose of this study was to compare the efficacy of lumbar epidural steroid injections for lumbosacral radiculopathy in individuals with obesity as defined by the CDC as BMI >30 as compared to non obese patients. The primary objectives were to examine the difference in pain relief, disability, and function using the Visual Analog Scale, Oswestry Disability Index, and PROMIS 29 profile in obese patients who underwent lumbar epidural steroid injections as compared to non-obese patients for lumbosacral radiculopathy at baseline and 3 months post injection.

**Results:**

This study is a retrospective analysis of patients who have undergone lumbar epidural steroid injections for lumbosacral radiculopathy at a single academic center. These patients underwent assessment using validated measurement scales at baseline and 3 months post ESI. Information from the UT Southwestern Spine Center outcomes registry and procedure notes were obtained to analyze pain, disability, functional outcomes from 2016 to 2021. 343 participants took part in the study. The results showed no significant difference in mean levels of ODI, VAS, or PROMIS-29 v1 0.0 scores when comparing values from baseline to 3 months in groups stratified by a BMI of <30 vs a BMI of greater than 30. Furthermore, there was no significant difference in mean levels of ODI, VAS, or PROMIS-29 v1.0 scores when comparing values from baseline to 3 months in groups further stratified by class of obesity: class 1 (BMI 30 to ​< ​35), class 2 (BMI 35 to <40), class 3(BMI 40 or higher).

**Conclusion:**

No significant differences were discovered in outcomes in patients undergoing lumbar epidural steroid injections when stratified by BMI or by BMI class. These results have significant clinical implications. Obesity is a known risk factor for the development of low back pain. ESI is a frequently employed method to treat this pain after conservative approaches are exhausted; however, ESI in obese patients typically are associated with more radiation exposure and increased technical difficulty. This study indicates that obesity does not greatly affect the efficacy of lumbar epidural steroid injections. Obesity should not be a strict contraindication to lumbar ESI. In addition, the results can guide clinicians in a well-informed shared decision-making discussion with obese patients regarding the risks and potential benefits of lumbar ESI.

## 1 Background/Introduction

Obesity, as defined by the CDC, is characterized by a body mass index (BMI) exceeding 30 [1]. Despite this simplistic definition, obesity is a complex and multifactorial disease process with wide-ranging implications including increased risk for disability, depression, type 2 diabetes, cardiovascular disease, cancer and mortality [2]. Over one third of the world's population is classified as overweight or obese [3]. Projections indicate that by 2030 approximately 38% of the world's population and 85% of the US population will be overweight or obese [4,5].

Low back pain is currently the leading cause of disability worldwide. Disability and cost secondary to low back pain is predicted to increase in the coming decades [6]. A meta-analysis revealed obesity as a risk factor for the development of low back pain with an odds ratio of 1.36 (95% CI, 1.18–1.57) [7]. An increase in pro-inflammatory cytokines produced by hypertrophied adipose tissue is theorized to be partially responsible for the increased prevalence of low back pain in obese patients in addition to increase mechanical stress caused by excess weight [8].

The etiology of low back pain is complex. For patients seen in a primary care setting >85% of patients will have nonspecific low back pain wherein a specific underlying condition cannot be identified; however, a majority of these cases resolve with supportive treatment [[10], [11], [12]]. Supportive treatment typically consists of nonpharmacologic interventions such as heat, massage and physical therapy and can include pharmacologic therapies.

Patients who do not respond adequately to conservative therapy can consider interventional procedures such as lumbar epidural steroid injection (ESI). The goal for lumbar ESI is to improve pain and function while reducing the need for spinal surgery. Obese patients undergoing lumbar spine surgery suffer greater intraoperative and postoperative complications including greater blood loss, longer operative times, and higher reoperation rates [26]. Given the increased risk of complications in obese patients, it is of importance to consider other measures prior to surgery. Despite the relative safety of lumbar ESI, serious complications can occur including paraplegia, quadriplegia, spinal cord infarction and stroke; thus, patient selection is key [27]. More common side effects of ESI include bleeding, cerebrospinal fluid leak, nerve irritation, post-injection headache, nausea, and transient blood glucose elevations [25]. A multi-center cohort study revealed that obese patients require longer fluoroscopy times during interlaminar lumbar ESI, thus exposing them to additional radiation [28]. Additionally, there is increased technical challenges in obese patients due to a greater body habitus requiring longer length needles and difficulty visualizing targets [29].

Upon literature review, three studies were identified comparing the efficacy of lumbar ESI in obese versus non-obese populations. The studies concluded there were significant reductions in pain and disability in both obese and non-obese patients with no significant differences between the two groups [20, 30, 31]. Two of these studies were performed in a population outside of the US [20, 30]. The remaining study was performed on a US population; however, it was a pilot study with a limited sample size of 24 patients [31]. The primary objective is to examine the difference in pain relief, disability, and function in obese patients who underwent lumbar epidural steroid injections as compared to non-obese patients. We hypothesize that there is no significant difference in pain relief, disability, and function in obese patients as compared to non-obese patients. Furthermore, this study is unique in that it stratifies patient outcomes by different classes of obesity.

## 2 Materials and methods

This study is a retrospective analysis of patients who have undergone lumbar epidural steroid injections for lumbosacral radiculopathy at a single academic center (University of Texas Southwestern Medical Center, Dallas, Texas, United States). These patients underwent assessment using validated measurement scales at baseline and 3 months post ESI, following institutional review board approval. Information from the spine center outcomes registry and procedure notes were obtained to analyze pain, disability, functional outcomes from 2016 to 2021. Patients were asked to fill out survey information during their clinic visits during this period. Informed consent was waived due to this being a retrospective study. The inclusion criteria includes all patients who underwent lumbar (interlaminar or transforaminal) or caudal epidural steroid injections with a diagnosis of lumbosacral radiculopathy and symptoms of both back and leg pain during the time frame of our study. Exclusion criteria includes those patients who were lost to follow up, had more than one procedure performed at the same time, or who only had back pain. Patients were stratified into two groups based on BMI: obese (BMI ≥30) and non-obese (BMI <30). Obese patients were further stratified by class of obesity: class 1 (BMI 30 to <35), class 2 (BMI 35 to <40), class 3 (BMI 40 or higher). The specific assessments utilized were the Visual Analogue Scale (VAS), the Oswestry Disability Index (ODI), and PROMIS 29 scores.

The Visual Analog Scale is a unidimensional measure of pain intensity. The higher the score indicates the greater severity of pain. The Oswestry Disability Index (ODI) is one of the most commonly used outcome measures for low back pain. It is a self-administered questionnaire divided into ten sections designed to assess limitations of various activities of daily living. Each section is scored on a 0–5 scale, 5 representing the greatest disability. The index is calculated by dividing the summed score by the total possible score, which is then multiplied by 100 and expressed as a percentage. Values ranging from 0 to 20% suggest minimal disability while values ranging from 40 to 60% suggest severe disability. The PROMIS-29 profile assesses pain intensity using a single 0–10 numeric rating item and seven health domains (physical function, fatigue, pain interference, depressive symptoms, anxiety, ability to participate in social roles and activities, and sleep disturbance) using four items per domain. It is a validated health-related quality of life instrument for various chronic conditions including back pain.

The primary outcomes in this study include improvements in Visual Analog Scale scores, Oswestry Disability Index scores and PROMIS 29 scores from baseline to 3 months post lumbar epidural steroid injection in the obese vs non-obese populations. The secondary outcomes include comparing mean levels of pain, disability, and function in patient's receiving lumbar epidural injections as stratified by specific class of obesity (Class 1, 2, or 3) at baseline to 3 months. Frequency counts were determined for all categorical measurements overall and by BMI category. Means and standard deviations were calculated for all time periods and for BMI categories. Means and standard deviations were calculated for each of 44 differences scores. Student t tests for paired observations were made for all difference scores. For those subjects with a BMI measurement, each difference score was compared by BMI category to determine if BMI affected that particular outcome. Analysis of variance (ANOVA) was conducted to analyze the differences among the means stratified by classes of obesity.

No adjustment for multiple comparisons was made except that statistical significance was set at 0.01 to decrease the probability of type I error.

## 3 Results

Of 386 eligible participants, 343 participants took part in the study (age M(SD) ​= ​64.25(14.48); Female N(%) ​= ​193(56.27); Non-Hispanic N(%) ​= ​308(89.80); Hispanic N(%) ​= ​25(7.29). 43 patients were excluded due to failure to follow up. Participant comorbidities are displayed in Table 1a. Table 1b depicts the patient demographics.

**Table 1a:** Patient comorbidities.

COMORBIDITIES N(%)	
HYPERTENSION	146 (42.57)
DIABETES	73 (21.28)
DEPRESSION	52 (15.16)
CANCER	47 (13.70)
ANXIETY	41 (11.95)
CONGESTIVE HEART FAILURE	12 (3.50)
HISTORY OF CEREBROVASCULAR ACCIDENT	11 (3.21)
CHRONIC OBSTRUCTIVE PULMONARY DISEASE	9 (2.62)

**Table 1b:** Patient demographics.

N (%)	
Gender	
Male	150 (43.7%)
Female	193 (56.3%)
Race	
White	258 (75.2%)
Black	54 (15.7%)
Other	31 (9.1%)
Ethnicity	
Non-Hispanic	308 (89.8%)
Hispanic	25 (7.3%)
Unknown	10 (2.9%)
Marital Status	
Married	219 (63.8%)
Single, widowed, or divorced	121 (35.3%)
Other	3 (0.9%)
Employment	
Full/part time/self employed	112 (32.7%)
Retired	176 (51.3%)
Not employed	45 (13.1%)
Other	10 (2.9%)

Table 2 depicts no significant difference in mean levels of ODI, VAS, or PROMIS-29 scores when comparing values from baseline to 3 months in groups stratified by a BMI of <30 vs a BMI of ≥30. The mean change in Oswestry Disability Index score was −5.74 in the BMI <30 group as compared to −7.33 in the BMI ​≥ ​30 group. The mean changes in VAS scores were −1.70 and −1.80 in the non-obese and obese group respectively. The PROMIS Pain score decreased by 1.57 in the non-obese and 1.67 in the obese. The PROMIS Satisfaction with Social Participation score increased by 0.78 in the non-obese and 1.55 in the obese. The PROMIS Sleep Disturbance and Sleep Related Impairment score decreased by 0.44 in the non-obese and 0.79 in the obese. The PROMIS Fatigue score decreased by 1.04 in the non-obese and 1.01 in the obese. The PROMIS Depression score decreased by 0.58 in the non-obese and 0.50 in the obese. The PROMIS Anxiety score decreased by 0.80 in the non-obese and 0.44 in the obese. Lastly, the PROMIS Physical Function score increased by 1.09 in the non-obese and 1.40 in the obese.

**Table 2:** Changes in mean levels of disability, pain and function in patients receiving ESI from baseline to 3-months stratified by BMI.

OUTCOME	N (BM ​< ​30)	N (BMI≥30)	M ​± ​SD BMI<30	M ​± ​SD BMI≥30	Δ M ​± ​SD	P-VALUE
OSWESTRY DISABILITY INDEX (ODI)	133	123	−5.74 ​± ​15.49	−7.33 ​± ​18.60	1.59 ​± ​17.06	0.46
VISUAL ANALOG SCALE (VAS)	154	147	−1.70 ​± ​2.92	−1.80 ​± ​3.23	0.10 ​± ​3.07	0.77
PROMIS-PAIN	158	148	−1.57 ​± ​2.62	−1.67 ​± ​2.63	0.09 ​± ​2.63	0.74
PROMIS-SATISFACTION	158	148	0.78 ​± ​5.76	1.55 ​± ​5.69	−0.76 ​± ​5.72	0.25
PROMIS-SLEEP	158	148	−0.44 ​± ​3.61	−0.79 ​± ​3.92	0.35 ​± ​3.76	0.42
PROMIS-FATIGUE	158	148	−1.04 ​± ​3.82	−1.01 ​± ​4.11	−0.03 ​± ​3.97	0.95
PROMIS- DEPRESSION	158	148	−0.58 ​± ​3.30	−0.50 ​± ​2.6688	−0.08 ​± ​3.01	0.83
PROMIS- ANXIETY	158	148	−0.80 ​± ​3.45	−0.44 ​± ​2.83	−0.36 ​± ​3.17	0.32
PROMIS-PHYSICAL FUNCTION	158	148	1.09 ​± ​4.16	1.40 ​± ​3.99	−0.30 ​± ​4.08	0.52

Table 3 depicts no significant difference in mean levels of ODI, VAS, or PROMIS-29 scores when comparing values from baseline to 3 months in groups further stratified by class of obesity: class 1 (BMI 30 to <35), class 2 (BMI 35 to <40), class 3 (BMI 40 or higher).

**Table 3:** ANOVA(Analysis of Variance) comparing mean levels of disability, pain and function in patients receiving ESI from baseline to 3-months stratified by class of BMI.

OUTCOME	P-VALUE
OSWESTRY DISABILITY INDEX (ODI)	0.89
VISUAL ANALOG SCALE (VAS)	0.97
PROMIS PAIN	0.46
PROMIS SATISFACTION	0.47
PROMIS SLEEP	0.78
PROMIS FATIGUE	0.36
PROMIS DEPRESSION	0.99
PROMIS ANXIETY	0.80
PROMIS PHYSICAL FUNCTION	0.73

Table 4 considers categorical data for outcomes of pain treatment the via the approach of Bogduk and Stojanovic [36]. We examined percent improvement in each of the BMI groups based on 30% improvement as a clinically important difference and generally found no significant differences. Interestingly. However, the data shows that improvement percentages were not approximately equal across BMI groups and that the heaviest group seemed to have the least improved percentage except for the PROMIS Fatigue value which is higher in the BMI ​≥ ​40 group compared to the BMI <30 group.

**Table 4:** Percent improved: 30% or more at 3 months.

Outcome	BMI <30	30 ​≤ ​BMI<35	35 ​≤ ​BMI <40	BMI ​≥ ​40	P-Value
OSWESTRY DISABILITY INDEX (ODI)	48/133 (36.1%)	28/74 (37.8%)	11/33 (33.3%)	6/16 (37.5%)	0.975
VISUAL ANALOG SCALE (VAS)	68/144 (47.2%)	45/84 (53.6%)	16/37 (43.2%)	6/18 (33.3%)	0.398
PROMIS Pain	67/156 (43.0%)	46/89 (51.7%)	12/38 (31.6%)	5/18 (27.8%)	0.092
PROMIS Satisfaction	25/155 (16.1%)	13/89 (14.6%)	5/39 (12.8%)	3/19 (15.8%)	0.959
PROMIS Sleep	25/156 (16.0%)	17/89 (19.1%)	7/39 (18.0%)	2/19 (10.5%)	0.808
PROMIS Fatigue	40/157 (25.5%)	24/89 (15.7%)	6/39 (15.4%)	6/19 (31.6%)	0.464
PROMIS Depression	27/157 (17.2%)	14/89 (15.7%)	7/39 (18.0%)	3/19 (15.7%)	0.987
PROMIS Anxiety	41/157 (26.1%)	16/89 (18.0%)	7/39 (18.0%)	1/19 (5.3%)	0.116
PROMIS Physical Function	15/158 (9.5%)	10/89 (11.2%)	3/39 (7.7%)	0/19 (0.0%)	0.476

## 4 Discussion

The results of this study mirror the results of prior studies. One small study conducted in Iran by Masoud et al. depicted no statistically significant differences in pain scores or disability between obese and non-obese patients with spinal disc herniation who underwent ESI [20]. In addition, a small pilot study (n ​= ​18) conducted by McCormick et al. revealed no significant differences in percent improvement of pain in patients receiving lumbosacral transforaminal ESI when stratified by BMI [31]. This study was unique due to a large sample size (n ​= ​343) whereas prior studies were limited by small samples. In addition, this study was comprehensive and evaluated disability, pain, and function simultaneously using VAS, ODI, and PROMIS 29 scales. This study also further stratified BMI by classes of obesity to delineate if there were significant changes depending on severity of obesity.

Although no significant difference was discovered in outcomes when stratified by BMI or by BMI class, these results have significant clinical implications. Epidural steroid injections are a frequently employed method to treat back pain after alternative conservative approaches are exhausted; however, ESI in obese patients typically requires longer fluoroscopy times and is more technically challenging [28]. This study indicates that obese patients do not have significant differences in outcomes after epidural steroid injections as compared to non-obese patients, regardless of severity of obesity.

Proposed mechanisms of action of epidural steroid injections include local inhibition of phospholipase A2 and inflammatory mediators, direct analgesic effects and reduced conduction in unmyelinated C fibers, and “washout” effect of inflammatory cytokines [[32], [33], [34], [35]]. No differences in outcomes in non-obese vs obese patients suggest that the proposed mechanisms of action of epidural steroid injections is effective enough to counteract the increased mechanical stress of obese patients.

### 4.1 Conclusions

There remains a debate whether lumbar ESI reduces the need for surgery in patients with sciatica [21]. Obese patients are prone to suffering greater intraoperative and postoperative complications from lumbar spine surgery [26]. If there are no significant differences in outcomes for obese vs non-obese patients undergoing lumbar ESI, it is prudent to explore this approach prior to surgery. Multiple systematic reviews and meta-analyses have depicted the efficacy of epidural corticosteroid injections for radiculopathy with immediate improvements in pain and function [21]. These improvements could enable obese patients to further participate in physical therapy as well as physical exercise to help reduce their weight and subsequently reduce their back pain.

Limitations of this study, which may limit the external validity of this study, include potential sampling bias as our study was composed of 89.8% non-Hispanic individuals. This may be due to disparities in healthcare access and is not necessarily representative of the general population. There is also a selection bias to consider with this study due to a loss to follow up of 43 patients. Finally, there is lack of homogeneity with 5 different physicians performing interventions over the course of the 5-year span of the analysis. It is difficult to determine the magnitude of impact that these limitations had on the results of the study.

Future directions from this study include performing a randomized control trials to further investigate the efficacy of epidural steroid injections in obese and non-obese patients. Furthermore, comparison of disability, pain and functional scores in patients receiving lumbar ESI could be stratified by comorbidities. Conversely, examining how epidural steroid injections effect obesity could be another source of future inquiry.

## Conflict of interests

The authors in this study have no competing or conflict of interests.

## Funding

This research did not receive any specific grant from funding agencies in the public, commercial, or not-for-profit sectors.

## Declaration of competing interest

The authors declare that they have no known competing financial interests or personal relationships that could have appeared to influence the work reported in this paper.
